# Taking a Break: The Effects of Partaking in a Two-Week Social Media Digital Detox on Problematic Smartphone and Social Media Use, and Other Health-Related Outcomes among Young Adults

**DOI:** 10.3390/bs13121004

**Published:** 2023-12-08

**Authors:** Paige Coyne, Sarah J. Woodruff

**Affiliations:** 1 Department of Public Health Sciences, Henry Ford Health, Detroit, MI 48202, USA; 2 Department of Kinesiology, University of Windsor, Windsor, ON N9B 3P4, Canada; woodruff@uwindsor.ca

**Keywords:** smartphone addiction, social media addiction, mixed methods, joint display, physical health, mental health, social health

## Abstract

Despite their increasing popularity, especially among young adults, there is a dearth of research examining the effectiveness of digital detoxes focused on restricting or limiting social media use. As such, the purpose of this exploratory study was to create and carry out a social media digital detox among young adults and evaluate its effectiveness with regards to smartphone and social media addiction, as well as several health-related outcomes. Additionally, the study also sought to obtain an understanding of participants’ experiences and perceptions regarding the digital detox via semi-structured exit interviews in order to improve and maximize the effectiveness of future social media digital detox interventions. Thirty-one young adults completed a two-week social media digital detox (preceded by a two-week baseline period and followed up by a two-week follow-up period), whereby their social media use was limited to 30 min per day. A series of one-way repeated measures analyses of variance revealed that a two-week social media detox improved smartphone and social media addiction, as well as sleep, satisfaction with life, stress, perceived wellness, and supportive relationships. Thematic analysis of exit interviews also revealed eight themes: feelings, effort to detox, adjustment period, the Goldilocks effect, screen to screen, post-detox binge, progress not perfection, and words of wisdom, all of which provide contextualization of the quantitative findings and valuable insights for future detoxes. In conclusion, the findings of this exploratory study provide initial support for the use of social media digital detoxes, suggesting that limiting usage can have beneficial effects with regards to smartphone and social media addiction, as well as many other health-related outcomes.

## 1. Introduction

As of 2023, smartphones are omnipresent in everyday life. They provide users with most (if not all) of the functions of a standard desktop computer but with the unprecedented freedom of mobility, allowing access to these functions whenever and wherever. As a result, worldwide usage has skyrocketed to approximately four hours of usage per day [1,2].

### 1.1. Positive and Negative Consequences of Smartphone Use

Given the pervasiveness with which smartphones have become integrated into modern society, their usage has been linked to a number of consequences, both positive and negative. With regards to positive consequences, smartphones allow users to make phone calls, send and receive text messages and emails, capture and display photos and videos, navigate to a destination, play games, surf the web, and access social media applications [3]. Hence, such features and functions allow users to communicate, connect, interact, and obtain information whenever they like [4,5,6,7]. However, despite their practicality and convenience, public and academic concern regarding the negative consequences of smartphones has mounted in recent years. Particularly, smartphones are said to have the potential to impair users’ health and well-being. For example, smartphone usage has been linked to impaired mental health (e.g., depression or anxiety; [8,9]), decreased self-esteem and well-being [10], and a number of other unhealthy lifestyle practices, such as disordered eating (e.g., meal skipping [11,12]), poor sleep [11,12], reduced physical activity and increased sedentary behaviour [13], and the use of drugs and alcohol [14], among others. Moreover, users are also more likely to engage in unfavorably viewed smartphone-related behaviours, such as phubbing (i.e., snubbing another individual in a social setting by paying attention to one’s phone instead of interacting with the person who is actually with them [15]), and develop nomophobia (fear of being without/not being able to use one’s smartphone [16]) or a digital fear of missing out (i.e., “fomo”; anxiety or fear of missing out on experiences their contemporaries are engaging in [17]).

Although many researchers urge the importance of not over-pathologizing smartphone use and highlight that many of the associations between smartphone use and negative outcomes are small [18], concerns regarding the negative impacts of smartphones (and their applications) continue to be discussed amongst greater society and by its individual members, so much so that many users are discussing their desires to revert to *brick* phones (i.e., those with limited functionality [19]) or spend time away from their smartphone [20], with many, somewhat ironically, sharing their journeys of disconnection on social media (which is typically accessed via smartphone). Additionally, many others are leveraging their smartphone’s built in (e.g., iOS Screen Time or Android Digital Well-Being) or third-party (e.g., Social Fever, App Detox) applications to better manage their smartphone time.

### 1.2. Digital Detoxes

To date, several different terms have been referenced to describe periods of time where smartphone users are not engaging with the device. As Radtke et al. [21] (p. 192) pointed out, terms such as “abstinence, break, disconnection, detox, time-out, and unplugging” have been used. However, in their systematic literature review, Radtke et al. [21] postulated that the term *digital detox* should be used as an umbrella term under which all the aforementioned terms could fall. They also suggested that the Oxford English Dictionary’s [22] current definition of digital detox is insufficient, highlighting that “*period of time during which a person refrains from using their electronic devices, such as smartphones, regarded as an opportunity to reduce stress or focus on social interaction in the physical world”* is incomplete. Specifically, they suggested that a digital detox occurs any time an individual refrains from (or reduces) their usage of *one or all their electronic devices* (e.g., detox from smartphone only and still use your other devices or detox from all devices) *or certain applications* (e.g., all social applications or a single social application, such as TikTok), branded media, special features, interactions, and/or messages. They also highlighted that the detox itself need be completed *voluntarily* and *intentionally*, with the purpose of *promoting health-related change* [21].

### 1.3. Social-Media-Specific Detoxes

Of all smartphone applications, social media applications, such as TikTok and Instagram, are among the most popular and time-consuming smartphone applications accessed by users, especially amongst younger generations who have grown up connected to the digital world [23]. Specifically, worldwide usage of social media, which are “internet-based applications that build on the ideological and technological foundations of Web 2.0 and that allow the creation and exchange of user-generated content” [24] (p. 60), has reached two hours a day, with most of this usage accomplished via smartphones [25]. Additionally, of all the applications available to smartphone users, researchers and larger society appear to be particularly concerned about negative consequences to users’ health that result from engagement with social media applications [21]. Thus, in addition to the considerable attention being paid to digital detox interventions restricting the use of the device (i.e., the smartphone), interest in more specific digital detox interventions, such as those that restrict the use of specific types of applications (e.g., social media applications), have garnered popularity. Particularly, given that smartphones have become an all but necessary component of everyday life, detoxing from social media instead of complete device abstinence or restriction may better balance the potential of obtaining improvements in health-related outcomes while still enabling users to continue, mostly uninterrupted, with daily life.

### 1.4. Social Media Digital Detoxes and Health-Related Outcomes

In a recent systematic literature review of intervention studies examining digital detoxing as the main behaviour change strategy, Radtke et al. [21] identified only 21 studies that had conducted digital detoxes focused on examining changes in health-related outcomes. Among these studies, the health-related outcomes investigated included sleep [26,27], life satisfaction [28,29,30,31,32,33,34], subjective well-being [28,29,30,32,33,34,35,36], anxiety [27,37,38,39], stress [27,33,34,40,41], depression [27,32,37], smartphone addiction [42], and social support [37]. Yet, Radtke et al. [21] noted contradictory findings (for example, Liao [27] reported a decrease in anxiety because of a smartphone detox, whereas Skierkowski and Wood [38] and Wilcockson et al. [39] found no effect) for nearly all the variables (excluding depression (Hunt et al. [37], Liao [27], and Tromholt [32] all saw improvements) and smartphone addiction (only measured by Ko et al. [42]) and identified several limitations and opportunities for future research. Notably, Radtke et al. [21] reported a lack of device-based measurements of smartphone and social media usage (i.e., lack of reliable and objective duration of usage data) and a lack of short- or long-term follow-ups. Moreover, they suggested that future research investigating social media digital detoxes should avoid focusing on the complete discouragement of smartphone and/or social media usage as this may not be realistic in everyday life, especially amongst younger generations who have grown up with both technologies. Rather, digital detoxes should be focused on encouraging users to develop healthy relationships with their smartphones and social media [43,44].

Among these studies, even fewer conducted social media digital detoxes (*n* = 13; [21]) despite their popularity amongst smartphone users [45] and their potential to positively impact health-related outcomes without disrupting everyday life. Specifically, social media digital detoxes have focused primarily on mental (e.g., life satisfaction, well-being, affect, boredom, stress, anxiety, and depression [28,29,37]) and social (e.g., connection, loneliness, social support, and pressure [36,37]) outcomes, and, again, mixed results have been reported. For example, with regards to life satisfaction, Hanley et al. [28] found no effect because of a 1-week social media digital detox, whereas Fiorvanti et al. [29] reported an Increase in life satisfaction after a 1-week Instagram digital detox, with Vanman et al. [33] reporting a decrease in life satisfaction after a 5-day Facebook digital detox. Thus, more studies examining mental- and social-health-related outcomes are needed.

Additionally, there is an even greater dearth of digital detox studies (none of which were social media, only digital detoxes) examining physical-health-related outcomes. For example, sleep has only been assessed by Dunican et al. [26], who limited usage of all electronic devices for 48 h and reported no effect on sleep quality, and Liao [27], who limited smartphone use for 2 weeks and reported improvements in sleep quality for those with anxiety and depression symptoms, once again showcasing the existence of contradictory findings. As such, additional inquiries into the effects a digital detox might have on sleep, and other physical health outcomes such as physical activity, sedentary behaviour, and eating behaviours, are warranted.

Finally, given that individuals might try some version of a digital detox because they may self-identify their use as being addictive or problematic, the lack of studies (Radtke et al. [21] only identified Ko et al. [42]) examining smartphone and/or social media addiction and whether taking a break would result in bringing about a positive impact is surprising and necessitates further investigation. Finally, among the existing digital detoxes, particularly social media digital detoxes, it is difficult to determine what is contributing to mixed findings studying the same health-related outcomes. However, it is evident to the authors of this study that there is a need to begin identifying the types of designs and characteristics (e.g., duration of detox, usage limits imposed) that are most effective.

### 1.5. The Present Study

The purpose of this exploratory study was to create and deliver a social media digital detox that addressed the limitations of previous detoxes (e.g., a lack of device-based measurements, lack of follow-up measurements, and unrealistic restriction instead of a reduction in usage) and explore its merits with regards to smartphone and social media addiction use and several health-related outcomes, including physical (physical activity, eating behaviours, and sleep), mental (life satisfaction, stress, and well-being), and social health (relationships) outcomes. Additionally, it was also desired to obtain a better understanding of participants’ experiences and perceptions of the digital detox to improve and maximize the effectiveness of future social media digital detox interventions. Specifically, the following research questions were asked:

1. Does a social media digital detox impact smartphone or social media addiction?
2. Does a social media digital detox impact physical, mental, or social health?
3. What are participants’ experiences and perceptions of their social media digital detox?

## 2. Methods

### 2.1. Philosophical Approach

The authors of this study assumed a critical realist approach, recognizing that reality is composed of three domains: the empirical (experiences that are observed and experienced), the actual (events, both observed and unobserved, that are generated or constrained by mechanisms), and the real (structures, powers, and mechanisms that produce or constrain events [46,47,48]). Thus, the authors acknowledge that, although only one true reality exists, individual interpretations and perceptions of this reality are shaped and influenced by perception, creating unique realities for all individuals.

### 2.2. Design

A mixed methods research approach was used, whereby quantitative and qualitative data were gathered and integrated to combine the strengths of both techniques [49]. An embedded experimental design (two-phase model) was employed whereby qualitative data (i.e., semi-structured exit interviews) were collected after a quantitative one-group pretest–post-test experiment (with two-week follow-up) was carried out [50]. In doing so, the qualitative data can be used to help explain and contextualize the intervention (quantitative) results (i.e., RQ 1 and 2) while also unearthing additional information regarding participants’ experiences and perceptions associated with completing the intervention (i.e., RQ3).

### 2.3. Intervention

**Figure 1:**
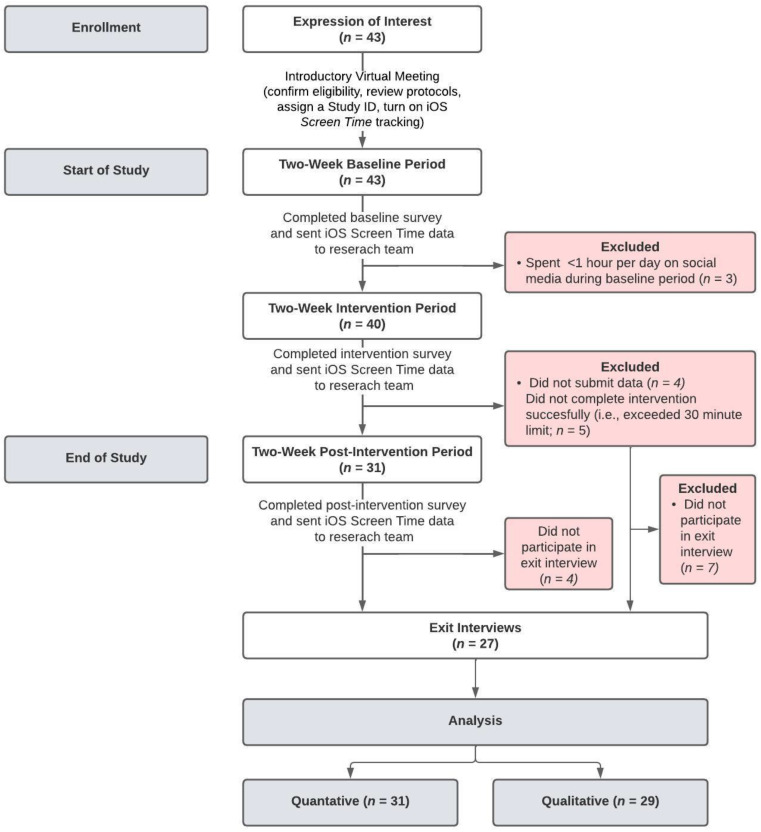
Study and participant flow.

A two-week social media digital detox, whereby the collective use of all social media applications (e.g., Facebook, Instagram, TikTok, Twitter) on participants’ smartphones was limited to 30 min per day, was carried out. This specific time limit was implemented in hopes that it would significantly reduce participants’ social media use on their smartphones but not be so restrictive that participants would be unable to complete the intervention successfully. The intervention period was preceded by a two-week period of normal use (i.e., no limits) to obtain a baseline of their overall smartphone and social-media-specific usages. Additionally, to see whether any changes that occurred during the intervention period remained after social media application limits were removed, a 2-week follow-up post-intervention period (i.e., limits were removed) was employed. All smartphone and social media application usage was objectively tracked using iOS’s(iOS 12) *Screen Time*, a built-in integration on all iPhones. Institutional research ethics board clearance (REB #22-116) was received prior to the start of recruitment and informed consent was obtained from all participants (see Figure 1 for participant flow).

### 2.4. Participants

Forty-three young adults were recruited from one department in a mid-sized university in Ontario, Canada via convenience sampling. Recruitment occurred via social media advertisement (i.e., departmental accounts, undergraduate, and graduate student society accounts) and email (i.e., mass emails to all students and in the monthly newsletter) in January 2023. Recruitment materials instructed interested students to contact a member of the research team via email. Upon contact, participants were provided with a letter of information and a copy of the consent form and asked to sign and return the consent form if they were still keen to participate, at which point an introductory meeting was scheduled.

Inclusion criteria were as listed: (a) undergraduate or graduate student in Kinesiology; (b) aged 18 to 30 years old; (c) iPhone user with *Screen Time* tracking turned on (or willing to turn it on for the duration of the study); (d) regular social media user (at least 1 h per day on social media applications); and (e) primary device for accessing social media was their iPhone. Participants also had to meet Radtke et al.’s [21] criteria of *voluntary* and *intentional participation* with the purpose of *seeking health-related change.* Participants were excluded from the study if they did not meet all inclusion criteria or if they were not willing to remove pre-existing *Screen Time* (or third-party application) limits to social applications for specified durations of the study (see Figure 1 for participant flow).

Of those that successfully completed the social media digital detox (i.e., were able to limit themselves to <30 min per day for the intervention period; *n* = 31), mean age was 21.16 (*SD* = 1.71 years; *range* = 18 to 26), with the majority currently completing undergraduate degrees (*n* = 25; 80.6%). More women *(n* = 21; 67.7%) than men (*n* = 10, 33.2%) participated in the study. Similar to Cowie and Braun [51], self-definition for ethnic/cultural origins information was utilized and resulted in the majority (*n* = 18; 58.1%) of participants identifying as White, while the remaining identified as South Asian (*n* = 5; 16.1%), Mixed (*n* = 4; 12.9%), Arab (*n* = 1; 3.2%), Chinese (*n* = 1; 3.2%), or Latin American *(n* = 1; 3.3%), with one participant (*n* = 1; 3.3%) opting not to disclose their ethnic/cultural origins.

### 2.5. Measures

At three separate timepoints (i.e., completion of baseline, intervention, and post-intervention periods), participants completed an online survey via Qualtrics. The surveys took, on average, 10–15 min to complete and contained questions pertaining to participants’ demographics (baseline only), smartphone addiction, social media addiction, physical activity, sedentary behaviour, sleep, eating behaviours, relationships, satisfaction with life, stress, wellness, and psychological well-being. Additionally, at each timepoint, participants were asked to retrieve data (i.e., total screen time and social application screen time) from their iPhone’s Screen Time integration for the previous two weeks.

#### 2.5.1. Survey

**Smartphone Addiction.** The Smartphone Addiction Scale—Short Version (SAS-SV; [52]) was used to measure participants’ smartphone addiction. The SAS-SV contains 10 items rated on a 6-point scale ranging from 1 (*strongly disagree*) to 6 (*strongly agree*). For example, “*Based on the past two weeks (i.e., baseline period), indicate the degree to which you agree with the following statements... Won’t be able to stand not having a smartphone*”. Total scores on this validated scale may range from 10 to 60, with higher scores indicating greater potential smartphone addiction. Previous studies using the SAS-SV suggest it has acceptable internal consistency and concurrent validity (a = 0.91; [53]). Cronbach’s alpha values for the current study (i.e., three different timepoints) ranged from 0.64 to 0.79.

**Social Media Addiction.** Bergen’s Social Media Addiction Scale (BSMAS; [54]) was used to measure participants’ social media addiction. The BSMAS rates 6 items on a 5-point scale, ranging from 1 (*very rarely*) to 5 (*very often*). For example, “*How often during the past two weeks (i.e., baseline period) have you... used social media to forget about personal problems?”.* Total scores may range from 6 to 30, with higher scores representing a greater risk of social media addiction [55]. The BSMAS has previously demonstrated good internal consistency (a = 0.88; [54]). Cronbach’s alpha values for the current study ranged from 0.71 to 0.80.

**Physical Activity and Sedentary Behaviour.** Participants’ physical activity and sedentary behaviours were assessed using three questions adapted from the World Health Organizations’ Global Physical Activity Questionnaire, version 2 (GPAQ; [56]). With regards to physical activity, participants were asked “*In the past two weeks (i.e., baseline period), on how many days did you do moderate- to-vigorous sports, fitness or recreational (leisure) activities?*” and “*In the past two weeks (i.e., baseline period), how much time did you spend, on average, doing moderate- to-vigorous sports, fitness or recreational (leisure) activities per day?*” to obtain duration (hours and minutes) and frequency (days per week) of recreational physical activity. Similarly, participants were asked “*In the past two weeks, how much time did you spend, on average, sitting or reclining per day?*” to quantify time spent engaging in sedentary behaviours during each time period. The GPAQ version 2 has shown acceptable reliability and modest validity in previous studies [57,58].

**Mindful Eating**. The Mindful Eating Questionnaire (MEQ; [59]) was used to evaluate mindful eating behaviours. The MEQ contains 28 items, rated on a 4-point scale of 1 (*Never/Rarely*) to 4 (*Usually/Always*), with 5 questions having an additional *Not Applicable* option. For example, “*I eat so quickly that I don’t taste what I’m eating.”,* with higher scores representing greater degrees of mindful eating. Subscale scores (i.e., disinhibition, awareness, external cues, emotional response, and distraction) are calculated as the mean of items, excluding “not applicable” responses. The summary score is the mean of the five subscales. Cronbach’s alpha values for the MEQ scales have ranged between 0.64 and 0.83 [59], and the MEQ also shows adequate criterion validity [59]. Cronbach’s alpha values for the current study ranged from 0.72 to 0.90.

**Sleep.** Five questions regarding sleep were adapted from the Canadian Community Health Survey (CCHS)’s Healthy Living rapid response module for the 2020 collection year [60]. Specifically, participants were asked about what time they usually fell asleep, how much time it took them to fall asleep, how often they woke up more than three times during their sleep, and what time they usually woke up during the previous two-week period. Participants were also asked to rate their sleep quality for the previous two weeks on a 4-point scale ranging from 1 (*poor*) to 4 (*excellent*).

**Life Satisfaction.** Satisfaction with life was measured using the Satisfaction with Life Scale (SWLS; [61]). The SWLS contains 5 items and uses a 7-point scale ranging from 1 (strongly disagree) to 7 (strongly agree). For example, “*So far I have gotten the important things I want in life.*” Total scores range from 5 to 35, with higher scores indicating greater satisfaction. Previously, the SWLS has demonstrated favorable psychometric properties, namely high internal consistency and temporal reliability [61,62]. Cronbach’s alpha values for the current study ranged from 0.88 to 0.91.

**Stress.** The Perceived Stress Scale (PSS; [63]) was used to measure participants’ stress levels. The PSS is made up of 10 items, scored from 0 (never) to 4 (very often). An example question would be, “*In the last month, how often have you felt that things were going your way?”.* For the purposes of this study, all items began with “In the last two weeks” instead of “In the last month” to reflect the study’s protocols (i.e., three timepoints). Total scores range from 0 to 40, with higher scores indicating higher perceived stress. Previous research suggests that the PSS has good internal consistency in university student populations, adequate test–retest reliability, and good validity [64]. Cronbach’s alpha values for the current study ranged from 0.69 to 0.80.

**Perceived Wellness.** Perceived wellness was measured using the Perceived Wellness Scale (PWS; [65]). The PWS assesses individuals’ perceived wellness across six life dimensions (i.e., psychological, emotional, social, physical, spiritual, and intellectual) and consists of 36 items scored on a scale ranging from 1 (*very strongly disagree*) to 6 (*very strongly agree*), with negatively worded items reverse-scored. For the current study, a modified 33-item version of the PWS (items 3, 9, and 21 from the original scale were removed) was used [66]. Total scores can range from 33 to 198, with higher scores representing greater perceived wellness. This modified version of the PWS has previously reported an internal consistency reliability estimate of 0.91 [66]. Cronbach’s alpha values for the current study ranged from 0.90 to 0.93.

**Supportive Relationships.** The Supportive Relationships subscale from the Mindful Self-Care Scale (MSCS; [67]) was used to assess the existence of and engagement with supportive relationships. The Supportive Relationships subscale contains 5 items. Item responses relate to the frequency with which participants engage in various behaviours. For example, “*I spent time with people who are good to me (e.g., support, encourage, and believe in me)*” with responses rated on a five-point scale, ranging from 1 (*Never*) to 5 (*Regularly*), and higher average scores indicating better self-care practices. Internal consistency reliability was 0.86 [67]. Cronbach’s alpha values for the current study ranged from 0.75 to 0.79.

#### 2.5.2. Total Screen Time and Social Application Screen Time

At the end of each two-week period (i.e., baseline, intervention, and post-intervention), participants were provided with instructions and asked to retrieve their daily total and social-application-related screen time (excluding Messages and FaceTime as these are not social media applications) from their iPhone’s *Screen Time* integration.

#### 2.5.3. Exit Interview

Participants’ experiences and perceptions of their social media digital detox were captured via optional semi-structured exit interviews (*n* = 27 who successfully completed the detox, *n* = 2 that did not finish successfully). All interviews were conducted by a single member of the research team. Although only one author conducted all interviews, informal peer debriefing with the other author took place throughout. Kallio et al.’s [68] framework for developing a qualitative semi-structured interview guide was employed (i.e., identifying that semi-structured interviewing is the appropriate data collection method, retrieving and using previous knowledge, formulating a preliminary interview guide and pilot-testing the guide, finalizing the complete (but still flexible) interview guide). All interviews were conducted virtually via Microsoft Teams. Each interview lasted about 10–15 min and consisted of questions pertaining to participants’ social media use prior to participating in the study, their experience partaking in the detox, and the impacts it may have had on their health. Participants were also encouraged to provide additional feedback on the logistics of the detox itself. When needed, additional probing questions were asked [69].

### 2.6. Data Analysis

#### 2.6.1. Quantitative Analysis

All data were analyzed using SPSS statistical package version 23 and JASP. Descriptive statistics were performed to acquire a profile of participants’ overall smartphone and social-media-specific smartphone usage. To address research questions one and two, 11 separate one-way repeated measures analyses of variance (RM ANOVAs) were conducted to determine whether there were statistically significant differences in smartphone addiction, social media addiction, and nine health-related outcomes (i.e., physical activity, sedentary behaviour, sleep duration and quality, mindful eating, relationships, life satisfaction, stress, and perceived wellness) over the course of a two-week social media digital detox intervention. Prior to conducting each RM ANOVA, testing for outliers, normality, and sphericity was completed. Only two assumption violations existed with the physical activity variable and perceived wellness. Namely, for physical activity, two extreme outliers were noted in both the pre- and post-intervention timepoints, and normality was violated at all timepoints. However, regardless of how outliers were handled (i.e., removing or modifying), issues with normality existed, and the results of the RM ANOVA remained comparable. As such, it was elected to keep the outliers and continue on with the RM ANOVA as it is quite robust to violations in normality. For perceived wellness, sphericity was violated, and results were thus interpreted using the Greenhouse–Geisser correction. For statistically significant models, uncorrected post hoc analyses were carried out. Additionally, Bayes factors (BF), using default priors, were computed for each analysis. Unlike frequentist statistics, which provides a measure of how unlikely the null hypothesis is, BF allow for the comparison of how likely null hypothesis (H_0_) is compared to an alternative hypothesis (H_A_), and vice versa [70], providing greater contextualization and the ability to interpret *p*-values greater than 0.05 (as highlighted by Wilcockson et al. [39]).

**Acknowledging Multiplicity.** The authors acknowledge that the current study conducted multiple tests of several outcomes and subsequent multiple comparisons at post hoc levels. Although correcting for multiplicity (e.g., through Bonferroni corrections) would protect against type I error (i.e., the probability of finding a significant result increases with more outcomes), it would also increase the chances for type II error [71]. Thus, given the exploratory nature of this study and that post hoc testing of unplanned comparisons is being conducted to serve as hypotheses generation for further investigations, the authors have opted to present the results without correcting for multiplicity [72,73]. However, readers are asked to keep in mind the exploratory nature of this study and are cautioned to carefully interpret the findings and not assign undeserved weight to them.

#### 2.6.2. Qualitative Analysis

All semi-structured interview audio recordings were transcribed verbatim, with the assistance of Microsoft Teams’ auto-generated closed-captioning. Reflexive thematic analysis, using an inductive–deductive hybrid approach [74], was conducted according to the steps outlined by Braun and Clarke [75], with focus on broad thematic patterning across the dataset by the same author that conducted all interviews. An inductive–deductive approach was selected as it ensures the voices of participants are heard and valued while simultaneously allowing previous research and theory to guide the analysis [74]. All themes were identified using a semantic approach whereby themes are derived from “the explicit meaning of the data and the analyst is not looking for anything beyond what a participant has said” [75] (p. 84). Finally, a code–recode procedure (with two weeks between coding and recoding) and reflexive journaling were employed to ensure the dependability of the data [76] and that the primary author performing the thematic analysis was cognizant of their own inherent biases and to mitigate their influence on the data [77].

#### 2.6.3. Integration of Datasets

After both quantitative and qualitative data analyses were completed, the data pertaining to research question two were compared to examine the (dis)agreement of the findings. A joint display of quantitative and qualitative findings summarizing the (dis)congruences within these findings was created.

## 3. Results

### 3.1. Quantitative Results

Descriptive statistics for objective smartphone and social media use across all three timepoints are presented in Table 1. In order to successfully complete the social media digital detox (i.e., stay within the 30 min per day limit), the participants reduced their social media usage on their smartphone, on average, by 77.7%.

#### 3.1.1. Smartphone and Social Media Addiction

The descriptive statistics for smartphone and social media addiction are available in Table 2. RM ANOVAs confirmed a significant main effect of time on smartphone and social media addiction (see Table 3). For smartphone addiction, significant differences were identified between all timepoints: pre-intervention and intervention [*p* < 0.001, 95%*CI* (4.679, 9.902), BF_10_ = 5966.46]; pre-intervention and post-intervention [*p* = 0.006, 95%*CI* (1.166, 6.189), BF_10_ = 7.388]; and intervention and post-intervention [*p* = 0.002, 95%*CI* (−5.827, −1.399), BF_10_ = 15.804]. For social media addiction, significant differences were only observed between pre-intervention and intervention [*p* < 0.001, 95%*CI* (1.517, 5.128), BF_10_ = 42.973] and between intervention and post-intervention [*p* = 0.015, 95%*CI* (−3.590, −0.410), BF_10_ = 3.089].

#### 3.1.2. Health-Related Outcomes

Descriptive statistics for health-related outcomes are presented in Table 2.

**Physical Outcomes.** There was no significant main effect of time on physical activity, sedentary behaviour, or mindful eating (Table 3). However, significant main effects of time regarding sleep duration and quality were observed. Specifically, for sleep duration, there were significant differences between pre-intervention and intervention [*p* = 0.020, 95%*CI* (−60.355, −5.451), BF_10_ = 2.446] timepoints and between pre-intervention and post-intervention [*p* = 0.049, 95%*CI* (−44.443, −0.73), BF_10_ = 1.195] timepoints. For sleep quality, significant differences between pre-intervention and intervention [*p* = 0.006, 95%*CI* (−0.763, −0.140), BF_10_ = 6.897] timepoints, as well as between intervention and post-intervention [*p* = 0.048, 95%*CI* (0.003 to 0.642), BF_10_ = 1.220] timepoints were reported.

**Mental Outcomes.** Significant main effects of time on satisfaction with life, stress, and perceived wellness were observed (Table 3). For satisfaction with life, the only significant difference was between pre-intervention and post-intervention timepoints [*p* = 0.010, 95%*CI* (−3.089, −0.459), BF_10_ = 4.509]. For stress, there were significant differences between pre-intervention and intervention [*p* = 0.005, 95%*CI* (0.849, 4.441), BF_10_ = 7.684] timepoints and between pre-intervention and post-intervention [*p* = 0.035, 95%*CI* (0.119, 2.978), BF_10_ = 1.584] timepoints. For perceived wellness, there were significant differences between pre-intervention and intervention [*p* = 0.002, 95%*CI* (−8.376, −2.140), BF_10_ = 20.437] timepoints and between pre-intervention and post-intervention [*p* = 0.016, 95%*CI* (−8.844, −0.962), BF_10_ = 2.929] timepoints.

**Social Outcome.** A significant main effect of time on supportive relationships was observed. There were significant differences between pre-intervention and intervention [*p* = 0.020, 95%*CI* (−0.331, −0.030); BF_10_ =2.442] timepoints and between intervention and post-intervention [*p* = 0.007, 95%*CI* (0.045, 0.265), BF_10_ = 5.784] timepoints.

### 3.2. Qualitative Results

Qualitative analysis relating to research question two resulted in the identification of eight themes. The themes are briefly discussed below and presented in Table 4, with example quotes.

The qualitative analysis suggested that the participants experienced a range of feelings while partaking in the detox (Theme 1). Particularly, participants often expressed that they enjoyed the detox, often experiencing feelings of relief and decreased pressure to maintain their social media connections and/or presence. Conversely, some participants did acknowledge that the detox brought up feelings of disconnection, from friends and family, as well as what was going on in the world during that time. However, many participants were pleasantly surprised that the detox was perhaps easier than they thought it would be (Theme 2) and that the timing of the detox was likely a factor. Namely, most participants were in the midst of midterm season and expressed that being busy made it easier to adhere to the detox.

Numerous references were also made to the challenge of adjusting to the detox (Theme 3), be it the disruption to a participant’s normal routine, filling the 10 min between classes, or getting over the shock of the 30 min limit during the first few days. However, despite these initial challenges, most participants were able to adjust their routines, find their groove, and stay within their half hour of usage, so much so that many participants suggested that half an hour was a sort of manageable sweet spot, where they could still engage with social media but not get caught scrolling for hours (Theme 4). Nevertheless, there was consistent acknowledgment that, while social-media-related screen time was reduced during the detox period, overall screen time remained relatively high, with many participants saying they used other applications (e.g., games or entertainment applications) or other screens (e.g., laptop) more than they previously ever did (Theme 5).

Upon completing the intervention period, a great number of participants disclosed that they overindulged in social media for a short period of time (Theme 6). Interestingly, although some claimed to have done so somewhat subconsciously, many suggested that they were aware of the bingeing behaviour they were engaging in, that it only lasted a few days, and that their overall awareness of their social media usage increased as a result of participating in a detox (Theme 7). Similarly, other references to small changes in behaviours, such as adopting modified limits or establishing periods of downtime, were frequently expressed, suggesting that detoxing may have resulted in some small but positive residual changes.

Finally, the participants shared many valuable suggestions for future detoxes, with particular emphasis being placed on making detoxes realistic, sustainable, and personalized to each user, where possible (Theme 8).

### 3.3. Integration of Datasets

A joint display of quantitative and qualitative findings for health-related outcomes is available in Table 5. It is important to note that, during qualitative interviewing, the participants were asked if they perceived any health-related changes in themselves as a result of partaking in the detox. As such, the participants focused on talking about health-related changes they experienced rather than discussing changes they did not experience.

At times, this display highlights the congruence between quantitative (frequentist and Bayesian) and qualitative data. For example, although the evidence was limited, there were quantitative data to support changes in stress across timepoints, which was supported by the qualitative data. In other instances, various incongruences are highlighted. For example, frequentist statistics suggest that the null hypothesis is rejected, but only with a small effect size, whereas the BF_10_ suggests there is strong evidence to support the alternative hypothesis, which itself is supported quite nicely by qualitative quotes provided by many participants. Moreover, this joint display highlights additional nuances in the data. For example, physical activity and mindful eating are two outcomes that statistically showed no change across timepoints. However, qualitative interviewing revealed many instances where participants experienced or perceived changes in these outcomes. In some instances, for example, with regards to physical activity, it appears as though the participants may have engaged in more low-intensity physical activity and experienced more focus when engaging in moderate-to-vigorous physical activity, neither of which were captured by the quantitative measures. Similarly, with regards to eating behaviours, many participants perceived change in some aspect of mindful eating (e.g., reducing the speed at which one eats), but not in a sufficient number of mindful eating characteristics to generate differences across timepoints on a quantitative scale.

## 4. Discussion

Despite their increasing popularity, a lack of research examining the effectiveness (and purpose) of digital detoxes is evident, with an even greater dearth of research regarding digital detoxes focused on restricting or limiting social media use. Moreover, although associations between digital detoxes and many health-related outcomes have been studied, many methodological limitations have been noted, and findings remain diverse. As such, the purpose of this exploratory study was to create and carry out a social media digital detox that addressed the noted limitations of previous detoxes (e.g., a lack of objective smartphone and social media use tracking or follow-up period and unrealistic expectation of completely restricted use) and evaluate the effectiveness of the detox with regards to smartphone and social media addiction, as well as a number of health-related outcomes. Additionally, and perhaps more importantly, the authors took a pragmatic philosophical stance and opted to complement quantitative findings with qualitative findings derived from participants’ experiences and perceptions of partaking in the detox.

### 4.1. Smartphone and Social Media Addiction

Consistent with previous studies that leveraged device-based [27,28,37,40,42,79] or self-reported measurements of smartphone or social media use [31,36], the participants in the current study experienced notable decreases in smartphone and social media use during the intervention period. However, this study’s use of a short-term two-week follow-up, which most previous studies have lacked, suggests that smartphone and social media usage may return to near pre-intervention levels within weeks, indicating potential backsliding.

Interestingly though, the detox resulted in reductions in smartphone and social media addiction for participants, which remained lower at two weeks post-intervention compared to the baseline. These results are similar to Ko et al. [42], who reported a significant reduction in smartphone addiction when smartphone use was limited. Yet, they also provided preliminary evidence that detoxing from social media on one’s smartphone has the potential to reduce not only smartphone but also social media addiction and that such reduction may be maintained, to some extent, for at least a few weeks.

### 4.2. Health-Related Outcomes

With regards to physical-health-related outcomes, the quantitative results of the current study (frequentist and Bayesian) suggest that the social media digital detox had no significant effects on physical activity (specifically MVPA), sedentary behaviour, or mindful eating. To the authors’ knowledge, no other study has quantitatively examined digital detoxes in the context of the aforementioned outcomes. This dearth of research is somewhat surprising given that high levels of smartphone and social media use have been linked to decreased physical activity, increased sedentary behaviour, and unhealthy eating habits in previous non-experimental studies [80,81], suggesting that detoxing from social media could influence these physical outcomes. Despite a lack of statistically significant changes to physical activity, sedentary behaviour, or mindful eating, participant interviews do suggest that additional contextualization of these findings is needed. In particular, many participants expressed partaking in more low-intensity physical activity (LVPA; e.g., walks or household chores), which may not have been captured statistically because the quantitative variable measured MVPA, not LVPA. Additionally, although no statistically significant effects on sedentary behaviour or mindful eating were observed, a few participants expressed that they spent more time on their feet or less time sitting and adopted better eating habits. However, it is likely that not enough participants experienced (or were aware of) a reduction in their sedentary behaviour and changes to their eating habits to elicit statistical significance. Regardless, additional research examining the impacts of digital detoxes on physical outcomes is warranted, with a need for researchers to careful select the measures they are using (e.g., LVPA vs MVPA).

Conversely, significant quantitative improvements in sleep duration and quality, which were maintained to some extent during the post-intervention period, were observed, contradicting the findings by Dunican et al. [26]. However, some incongruence between frequentist and Bayesian interpretations was noted for sleep quality, with frequentist suggesting a small effect and Bayesian interpretations suggesting greater (i.e., moderate) evidence for the alternative hypothesis. Regardless, these findings are in line with experimental research by Liao [27] and many other non-experimental studies [82], which provide evidence of an inverse relationship between smartphone or social media use and sleep. Similarly, the study’s quantitative findings draw considerable qualitative support and confirmation, with many participants explaining that they fell asleep earlier and experienced better-quality sleep.

When trying to understand why participants experienced statistically significant improvements in sleep but no other physical activity outcomes, the authors of the current study postulate that the participants had fewer options with which nighttime screen time could be replaced (e.g., *I went to bed earlier every single night because I was like, well, there’s nothing else for me to do*) compared to daytime screen time (e.g., more options to pick from, such as studying or going on another screen). Additionally, given that the participants were sourced from a kinesiology department, it could be that the participants already had good and established routines with regards to their physical activity but not their sleep.

With regards to the mental-health-related outcomes, changes in satisfaction with life, stress, and perceived wellness were observed, and supported by qualitative findings, with participants often noting reductions in negative feelings and stress, as well as increases in positive feelings, productivity, and confidence. Similar to Hinsch and Sheldon [31], Fioravanti et al. [29], and Tromholt [32], who each conducted detoxes of varying lengths of Facebook or Instagram, the participants reported small but significant increases in life satisfaction at the end of the post-intervention period compared to the pre-intervention period. Conversely, these results contradict those of Hall et al. [30] and Hanley et al. [28], who found no effects, and Vanman et al. [33] and Vally and D’Souza [34], who observed decreases in satisfaction. When examining stress, the findings from previous research are mixed, with a few studies reporting no effect of a digital detox on stress [33] and many others reporting notable decreases [27,40,41], with the later in congruence with the findings from the current study. However, discrepancies between previous studies reporting reduced stress and the current study exist with regards to magnitude, with previous studies observing medium to large effect sizes compared to the current study’s small effect size (frequentist) and moderate evidence for support for the alternative hypothesis (Bayesian). Finally, similar to Brown and Kuss [79], who reported increases in mental well-being as a result of a 7-day social media detox, the participants in the current study experienced improvements in their perceived wellness. Yet, as has been noted with other outcomes, the size of the effect or strength of the evidence supporting this result is questionable, with frequentist interpretations being small compared to stronger Bayesian interpretations.

A small but significant improvement in the study’s only social outcome, supportive relationships, was observed. These findings received some support from participant interviews, with several participants suggesting that they inherently spent more time interacting with members in or outside of their household. However, these findings are in opposition to the social media digital detox studies by Hunt et al. [37], who found no effect on social support, and Stieger and Lewetz [36], whose participants reported greater social pressure during their detox.

### 4.3. Experiences and Perceptions of Participants

In addition to using exit interviews to contextualize quantitative findings, it was important that this study be pragmatic and utilize these interviews to better understand participants’ experiences and perceptions of the social media detox, in hopes of providing suggestions for future social media detoxes. As a result, the authors were able to better understand how participants felt throughout the study, the effort it required for participants to detox, areas of challenge, obtain input on detox logistics, and identify areas for future improvement.

First, the authors assumed that the detox would be perceived to be difficult, and even unrealistic, for many individuals. As such, they anticipated (and experienced) difficulties recruiting. However, to the surprise of both the research team and the participants, two of the most commonly shared feelings associated with detox participation were enjoyment and relief, with many participants stating that the detox was easier than they thought it would be (Themes 1 and 2) and citing a range of positive feelings associated with their participation. Nevertheless, some participants did report feelings of disconnection and loneliness (Theme 1), which have previously been reported quantitatively [34,35]. Very few participants experienced these negative feelings to the extent that they terminated their participation or unsuccessfully completed the detox, with the majority of the participants indicating that they thought the study’s 30 min limit was challenging but doable. Thus, although nearly all, if not all, the participants said they experienced a period of adjustment (Theme 3; i.e., where they had to adjust to the detox’s reduced social media usage), most were able to overcome the trying first few days and successfully adjust their daily routines to reflect their reduced social media access.

Interestingly, many participants shared that their limited access to social media increased their perceived boredom during the intervention period, noting that small windows of time between classes or meetings were especially challenging. Such findings are consistent with Brown and Kuss [79], whose participants anticipated (before starting the intervention) that a reduction in social media usage would result in increased boredom, and Stieger and Lewetz [36], who quantified that a week-long social media detox led to increased boredom amongst participants. Again, similar to Brown and Kuss [79], the participants in the current study found themselves substituting social media use with other forms of screen time (Theme 5). Based on participants’ explanations, it is thought that this substitutive behaviour may not always occur consciously given the habitual nature with which younger generations engage with technology, especially for entertainment [79].

Additionally, as Radtke et al. [21] reported, only a limited number of digital detox studies have included follow-up measurements [36,37] of their outcome variables. As such, this exploratory study fills a significant gap in the literature by conducting post-intervention testing two weeks after the conclusion of the detox. Although qualitative accounts from participants suggest that many excessively indulged (i.e., binging) in social media for a short duration immediately following the intervention (Theme 6), comparisons of quantitative means pre- and post-intervention indicate that both addiction and all the health-related outcomes studied showed positive or neutral improvement. Thus, at minimum, participating in the detox did not negatively impact any quantitative outcomes amongst the participants in the current study, and any improvements made during the intervention period were maintained at least to some extent. Moreover, the qualitative findings from the exit interviews support the quantitative improvements observed during the intervention period and maintained during the post-intervention period. Namely, participants acknowledged that they became more aware of their social media habits and have already taken small steps (e.g., modifying limits or establishing periods of down time) to regulate their usage.

Finally, the participants provided several practical suggestions, based on their experience partaking in the detox, that could be incorporated into future detoxes to increase their overall efficacy and, perhaps more importantly, participant buy-in. Generally, these suggestions can be grouped into four categories. First, although most participants noted that limiting social media use to 30 min a day was difficult but reasonable, many acknowledged that, if they were to continue restricting their usage, they would opt for slightly more time. Thus, there is potential cause to conduct detoxes that are slightly less restrictive (e.g., 45–60 min of social media use per day) and to compare their effectiveness and buy-in to those of shorter durations. Moreover, one participant did point out that, instead of applying a one-size-fits-all approach to detox (i.e., all participants reduce their usage to the same amount of time), personalized limits (e.g., reducing each participant’s use by a specific percentage) could be more impactful and result in more buy-in from heavy users. Second, similar to Brown and Kuss [79], it was suggested that encouraging or enforcing participants to limit their social media application notifications could assist participants to complete the intervention period. Specifically, doing so would create less temptation to access social media simply just to see what caused that notification. Third, instead of restricting usage of all social media applications, many participants noted that certain applications are more destructive than others (e.g., many mentioned TikTok) and that limiting only those that were viewed negatively would be more realistic and, again, be easier for heavy users. Finally, during the intervention, some participants said that they opted to delete or sign out of some or all of their social media applications to reduce temptation, with several others suggesting that, in hindsight, the detox would have been easier if they had done so as well.

## 5. Limitations

Despite this exploratory study’s numerous strengths (e.g., objective smartphone and social media use tracking, the inclusion of a two-week follow-up period, and the choice to limit to totally restricted usage), it is not without limitations. With regards to study design, the following limitations are noted: use of a one-group pretest–post-test experimental design with follow up, non-objective measurements of outcome variables, and the inability to control for participants’ use of social media on other devices. Future studies could benefit from randomized controlled trial designs with a control group and objective measurements of not only smartphone and social media use among participants but also of targeted outcome variables and to control for participants’ use of other devices, if possible. Future researchers should also bear in mind that participants may consciously or subconsciously alter their social media usage during the baseline period in preparation for the reduced usage during the actual intervention period. To avoid this, limiting recruitment to only participants who already have *Screen Time* turned on and using their previous two weeks of data (or at least comparing the newly collected baseline data to the prior two weeks) could help to increase baseline data validity.

Additionally, although inclusion criteria were created to ensure that participants were regular users of social media (i.e., more than one hour per day, on average, during the pre-intervention period), descriptive statistics suggest that most of the participants in the study would not be classified as heavily addicted to their smartphones or social media. As such, the results of this study may not be generalizable to such populations, and future studies would benefit from trying to recruit such types of users. With regards to recruitment and participants, these additional limitations are acknowledged: convenience recruitment of university students, small sample, and exclusion of non-iPhone users (i.e., Android users). Although the previously mentioned limitations were unavoidable due to reasons related to feasibility and a desire for consistency in measurement, it is noted that these decisions restricted the individuals that qualified for participation and limit the generalizability of the study’s results. Finally, it is also worth again acknowledging that the authors opted not to correct for multiplicity. As such, the reader is reminded to interpret the quantitative results with caution (i.e., consider not just the *p*-values but also the effect sizes, confidence intervals, and BF_10_) and that these results should be used to encourage further research of the outcome variables.

## 6. Conclusions

The findings of this exploratory study provide some support for the use of social media digital detoxes. Specifically, although its findings should be considered carefully due to its exploratory nature, the results of this study suggest that limiting social media to half an hour a day for a two-week period may have beneficial effects with regards to smartphone and social media addiction, as well as many other health-related outcomes. Additionally, this study’s pragmatic approach and mixed methods design also provides a number of valuable insights for future researchers looking to design detoxes that are both realistic and effective.

## Figures and Tables

**Table 1:** Descriptive statistics for smartphone and social media use captured via iOS Screen Time.

Measure		*M*	*SD*	*Range*
Smartphone use (minutes)				
	Pre-intervention	312.35 ^a,b^	120.85	142 to 664
	Intervention	237.00 ^a,c^	121.25	100 to 665
	Post-Intervention	290.87 ^b,c^	102.94	148 to 548
Social media use (minutes)				
	Pre-intervention	129.81 ^d^	58.68	64 to 306
	Intervention	24.58 ^d,e^	5.21	7 to 30
	Post-Intervention	117.26 ^e^	53.67	29 to 231

*Note*. Statistical differences observed between the same letter; ^a^ *p* < 0.001, ^b^ *p* = 0.027, ^c^ *p* < 0.001, ^d^ *p* < 0.001, ^e^ *p* < 0.001.

**Table 2:** Means and standard deviations of outcome variables at all intervention timepoints.

Outcome		Pre-Intervention		Intervention		Post-Intervention	
		*M*	*SD*	*M*	*SD*	*M*	*SD*
Addiction	Smartphone addiction (10 to 60) ^a^	29.39	6.01	22.10	5.46	25.71	6.47
	Social media addiction (6 to 30) ^a^	14.94	3.75	11.61	3.33	13.61	4.13
Physical	Physical activity (minutes per day) ^a^	49.70	45.42	49.15	34.92	50.58	59.58
	Sedentary behaviour (minutes per day) ^a^	445.00	142.88	435.97	135.66	447.10	116.73
	Mindful eating (1–5)	2.32	0.35	2.29	0.31	2.28	0.38
	Sleep duration (0 to 24 h)	7.73	1.11	8.27	1.24	8.10	1.27
	Sleep quality (1 to 4)	2.58	0.72	3.03	0.80	2.71	0.74
Mental	Life Satisfaction (5 to 35)	23.94	6.44	24.90	6.12	25.71	5.78
	Stress (0 to 40) ^a^	18.39	4.61	15.74	4.58	16.84	4.88
	Perceived wellness (33 to 198)	140.39	18.16	145.65	18.05	145.29	19.21
Social	Supportive relationships (1 to 5)	4.14	0.65	4.32	0.56	4.17	0.55

*Note*. ^a^ indicates items where higher scores represent less-desirable outcomes.

**Table 3:** RM ANOVA statistics for study variables.

Outcome		*F* (2,62)	Exact *p*	ω^2^	ω^2^ Interpretation	*BF*_10_	BF Interpretation ^1^
Addiction	Smartphone addiction	18.442 ***	<0.001	0.189	large	39,275.319	Extreme evidence for H_A_
	Social media addiction	8.460 ***	<0.001	0.105	medium	71.561	Extreme evidence for H_A_
Physical	Physical activity	0.020	0.980	0.000	-	0.097	Strong evidence for H_0_
	Sedentary behaviour	0.140	0.870	0.000	-	0.110	Moderate evidence for H_0_
	Mindful eating	0.391	0.678	0.000	-	0.131	Moderate evidence for H_0_
	Sleep duration	4.067 *	0.022	0.026	small	2.180	Anecdotal evidence for H_A_
	Sleep quality	4.579 *	0.014	0.047	small	3.500	Moderate evidence for H_A_
Mental	Satisfaction with life	4.407 *	0.016	0.011	small	2.755	Anecdotal evidence for H_A_
	Stress	5.792 **	0.005	0.042	small	7.726	Moderate evidence for H_A_
	Perceived wellness	6.934 **	0.005	0.014	small	17.134	Strong evidence for H_A_
Social	Supportive relationships	3.875 *	0.026	0.013	small	1.879	Anecdotal evidence for H_A_

*Note.* *** *p* < 0.001, ** *p* < 0.01, * *p* < 0.05; ^1^ according to Lee and Wagenmakers [78].

**Table 4:** Themes from qualitative data.

Theme and Definition	Categories and Definitions	Example Quotes
*Theme 1—Feelings*: emotions felt because of detox	*Enjoyment*: participants said they felt good during the detox and liked how it was making them feel	I felt generally like good. (06) I actually like really enjoyed it like the break like I did actually feel like it was like a detox and like I don’t know if that’s like what I needed, but like I felt like it was very beneficial to me. (22) it was great for me, like I didn’t miss it as much as I thought I would. (28) I actually really enjoyed it. (29)
	*Relief*: participants communicated that they felt less obliged to maintain their social media presence, resulting in positive mood and reduced stress	[I was] relieved because there was a lot like, there were a lot of messages. I didn’t want to answer and that kind of had to excuse to not answer them. (03) I don’t feel pressured to participate because I have this excuse saying, like, you know, like, I actually do have a limit. (08) It was nice to not have to like, not feel like I had to be on my phone if I was doing nothing. (12)
	*Disconnection*: participants felt their disengagement from social media hindered their connection to people online and/or their ability to stay up to date with the happenings of the world	Yeah, like, felt a little disconnected from them if that makes sense (03) So, like I mentioned for like with my coworkers, right? I wasn’t like involved into that group chat. So, I feel like I was kind of feeling left out because I wasn’t caught up to date on like everything that they were talking about all the time. (08) I was behind on the latest gossip on pop culture. I think that would be something […] people had to update me on things in my social interactions instead of me being like, *did you see what they posted*? (18)
*Theme 2—Effort to detox:* effort required to partake in detox	*Easier than anticipated*: participants expressed that the detox was easier than they initially thought it would be	I was actually surprised that I wasn’t missing it [social media] more. (05) It was not that hard. I won’t lie. I think I actually enjoyed it. (16) Definitely wasn’t as hard as I thought it was going to be (22) I thought it would be difficult. It actually wasn’t as hard as I thought it would be. (39)
	*Being busy helps*: participants suggested that the detox was easier when they were busy compared to when they had less to do	It was definitely a lot easier to forget about my phone during that time period (week two of the intervention) because I had so much more going on than I did the first week (week one of the intervention). (01) But I found that I was quite busy during those weeks I found so it was super easy during those times to avoid it. (27)
*Theme 3—Adjustment period:* period required to adjust to restrictions of detox	*Change in routine*: participants said the detox disrupted their daily routine (e.g., morning or nighttime routine), creating some discomfort, at least initially	I think like my only thing is that my daily routine is, I’m pretty busy for most of my day. And then I reach like six seven o’clock and then I’m at home and I’m just sitting around, and I don’t have anything to do until I go to bed. I think the only challenging part was I used to just scroll on social media and stuff […] and so having to take out using my phone during that time and then filling it with other things was like a little bit of a hassle as first. (12) A little frustrating sometimes because I was going against my typical routines. I usually wake up, go downstairs, make my coffee, put on the news, and sit with my dog for like an hour just chilling. Which I’m on my phone checking multiple social medias, like doing that type of thing and while watching the news simultaneously. But like it disrupted that routine of just, like, chilling. (18) It was like getting out of my kind of zone of like what I usually do. Just lay here [in bed scrolling] for like an hour before [sleeping]. (12)
	*Getting over the hump*: participants expressed that the first few days of the detox were the most challenging but that it became easier in subsequent days	It was a little challenging, just at the beginning, just to kind of transition into [the detox] after like 2 weeks of just normally like using social media. (03) I’m not going to lie the first few days [of the detox] was kind of hard. (26) As it went on, yeah, it just became easier. (07) Yeah, it was challenging in the beginning, like I said, but once I kind of got over like adapted to it is when it kind of got easier. (14)
	*Small pockets of time*: participants struggled to fill small periods of time (e.g., 5 min) between back-to-back commitments	Just the, you know, the 15 min before an appointment… I was at the doctor’s office in a waiting room, and I don’t have my social media to scroll through. So, you are kind of just sat there like, *OK, I’m just going to sit*… so I that I think those were the most disruptive. (18) I felt kind of like I went back five years and had to figure out how to like, fill pockets of time with like reading the news and reading books or trying to bring a book with me, which I used to do growing up every time I go to a doctor’s office, I always brought a novel but I don’t really do that anymore, so it was just like random pockets of time trying to fill. (4)
*Theme 4—The Goldilocks effect*: the parameters of the detox were optimal	N/A	I thought it was kind of the perfect amount, like I didn’t need anymore. (05) Thirty minutes, it’s like kind of a good, happy medium. (07) I was still able to, like basically do everything that I normally do, just not waste as much time on it as normal. (11) It was like a sweet spot. That was like, manageable. (15) Without a shadow of a doubt, less time would have been tons more difficult, (01) It was not impossible for me to complete like I felt like I had enough time to, you know, check whatever is going on, see if anyone messaged me. And get that quick little dose of quiet time. (08) I think any less would have been maybe a bit too much of a cut. (19) It kind of allowed me to do things I wouldn’t normally do, just not like abuse that and go over it and just keep scrolling through it for no reason. (11)
*Theme 5—Screen to screen*: replacing social media screen time with other types of screen time	*Switching applications*: participants expressed that they spent more time than they normally would on non-social media applications on their smartphones during the detox	I did play a lot of (virtual) chess in those two weeks, (01) I started playing Sudoku. (03) I played Candy Crush, (12) So, I was like *oh, I’ll just read the news app*. […] Putting my screen time and other areas that wasn’t social media like I’m like, this isn’t going to stop me from being on my phone, but it’s stopping me from being on certain things. (18) So, my like redirects were Indeed. I was just scrolling through jobs because I just wanted to scroll and look. (18) I started watching Netflix a lot and YouTube videos, just because when I click on Instagram it’s like oh, you got two more minutes. (26)
	*Switching screen*: participants said that they spent more time than they normally would on other devices (e.g., laptop, tablet) during the detox	Like when I’m on my, like, study break, when I usually grab my phone, I’d like look up the most random things on Google on my laptop. (06) I would spend it doing more mindless things on like a different device […] I fell down the Wikipedia loophole so many times where I would just, you know, Google something and then I just like, I spent so many hours on the Internet just looking at random things. (11) I played video games sometimes too. (34)
*Theme 6—Post-detox binge*: excessive indulgence in social media after completing the intervention period	N/A	I definitely like binged like the first couple days, (04) I felt like I, like, almost like retaliated subconsciously by like using it a little more. (05) Uh, the first couple days I definitely binged out because I felt like I just went through like all my social media and just got a deep dive of everything and anything. (08) I was on kind of like trying to keep up or, like, backtrack on a lot of that stuff. So, I found myself scrolling and then sending videos in response and like things like that on social media. (12) I think I had a bounce back effect. It was like *oh I have access to it* again. Let me just like scroll aimlessly and like have no self-control for a couple days, (18) Yeah, I would say for the first probably three days, I binged, and I was like it was honestly just TikTok, (28)
*Theme 7—Progress not perfection*: focus on small, achievable steps in the right direction, not perfection	*Awareness*: participants acknowledged that they were more aware of their social media habits on their smartphone after completing the detox	I’m more aware of, you know, how much time I’m actually spending on my phone. And so, I do think about it a lot more now when I pick up my phone or I’m on social media. […] Where before, I would never think about that. […] But I do think I consciously think about it more now. (08) I felt like, Dang, I’m spending too much time now during this and stuff like I’m a bit more conscious now. (16) So, I felt like I am able to just cut a scroll…I felt like it [the intervention] changed my thinking a little bit about social media and like my usage. (22) Maybe I am more conscious of it. Like when I go on social media or when I when I get to a point when I’m scrolling and it’s like okay*, what am I doing like this headline is stupid. I’m going to close the app*. (29) And like I think it was interesting because it was like I felt overwhelmed before [the intervention], felt a lot calmer during [the intervention], and then afterwards I went right back to feeling overwhelmed again. And I’m like*, am I really that busy of a person or am I just like using my time really poorly?* So yeah, a lot of opportunity for reflection, I guess. (31) I’m thinking about just putting another timer on because I feel like I was a lot more productive than I am now. But like, I’m also going through burnout. So, I don’t want to completely say *ohh the social media timer completely changed my life, revolutionized everything but like*… I think I need to go back to that. (13)
	*Modified limits*: participants expressed a desire to continue limiting their social media usage but with modified limits	And honestly, I want it to be something we implement into, like our daily lives. Like maybe an hour or so, because I think spending like hours upon hours on your phone is definitely not like the best. Um, so having that little reminder of, *hey, you’ve spent this much time on social media today.* It’s kind of like a wake up. (15) Setting like an hour limit during the summer. (28)
	*Down time*: participants expressed that the detox has encouraged them not to access social media on their smartphone while they are completing other tasks	And then you’re now like, I don’t use my phone as much. I just put it to the side and study. (26) I still kind of fostered good, like, just little hobbies and productive tips during study that I still implement, because I found like, benefits to that. (28) Especially at the gym, cause between sets, I’ll just jump on. I’ll just go on my phone between sets. It’ll be 30 s, whatever. And then I’m like, scrolling for five minutes. I’m like, *I just wasted so much time just on for no reason*. So, when I was at the gym, I was thinking *I have 30 min a day*. You’re not going on my phone for social media at the gym, so I really liked that, and I’ve started doing that even after the limits went off. (34) I guess there are some things that I could like take, again, like my workouts were much like I, they were much more focused, so I guess I could do it like that and I could just limit my use during specific times of the day. (11)
*Theme 8—Words of wisdom*: considerations for future detoxes	*Realistic limit/reduction*: participants suggested that slightly more time (than 30 min) on social media would be more realistic and to consider personalized reductions instead of a one-size-fits-all approach	So, I think all around like if I were to set a limit myself, if I would start to get into that, maybe I’d do like 45 to an hour. (06) Personally, I think I like an hour. So, you have a little bit more buffer room. I don’t. I would hope that I wouldn’t use the hour everyday but was having that little bit of extra space that if I one day like I was texting people a bit more, I had that room. (15) I guess you could argue like relatively like if someone is like a 5–6 h/day social media person, you knock him down to two hours, like I guess relatively speaking that’s a reduction. (39)
	*Notifications*: participants suggested that detox compliance might increase if participants turn off their social media notifications to avoid temptation	[…] where they’re notifications are going way too fast and like it also this might be something of consideration, like I usually have all my notifications off anyway for all social media. So even if I were to get a notification my phone wouldn’t buzz […], it’s just a badge. (18)
	*Identify the most problematic*: participants suggested focusing the detox on certain apps, specifically those deemed most problematic by the user	I think the only thing I would limit would be TikTok again, just because it has so much new content, you lose track of time when you’re scrolling. I wouldn’t limit any other social media, but TikTok, I think that one is the only one that’s more problematic. (18) I’m planning on deleting a couple of apps right now. […] I have been thinking about the deleting TikTok. (34)
	*Delete or deactivate:* participants suggested deleting or deactivating social media applications during the detox to reduce temptation	Yeah, I think if anything, I’d probably do like what I’ve done the past like just deactivate my account just to get off it completely. (19) I think deleting the apps would have been more effective or even signing out of them. (29)

*Note.* Bracketed numbers (e.g., (13)) represent unique numbers assigned to participants to maintain their anonymity.

**Table 5:** Joint display of quantitative and qualitative findings regarding health-related outcomes.

Outcome	Quantitative Findings (Frequentist and Bayesian)	Qualitative Findings (Quotes from Participants about Health-Related Changes They Noticed as a Result of the Detox) ^1^
Physical Activity and Sedentary Behaviour	Frequentist: Maintain H_0_ BF: Strong and moderate evidence for H_0_	I did go on a couple of walks and honestly, it was like those random periods of time when usually you would scroll that I was more like tempted to just get up and move around. (04) When I was working out, I found myself like, not more focused, but well, I guess more focused, like less distracted during my workouts […]. (13) I took my dog on more walks. (15) But I did like more stuff around the house, like didn’t leave my laundry for, you know, days before like folding it or like doing other, like, cleaning up a little bit, like not procrastinating certain things around the house or getting some errands done that I was putting off for a while, just filling that that I would normally sit and just like, look at my phone. (07)
Sleep Duration and Quality	Frequentist: Reject H_0_ but small effect size BF: Anecdotal and moderate evidence for H_A_	Right. It did have an effect I was sleeping a little bit earlier and better. Like I wasn’t waking up throughout the night and I think it was I woke up like not angry, like woke up refreshed. (13) I think I would go to bed like I would fall asleep quicker. Because I wouldn’t scroll that much before. (16) I went to bed earlier every single night because I was like, well, there’s nothing else for me to do. (27) I feel like I almost slept better because I wasn’t going on my phone like right before bed because usually by the time I hit a bedtime like my limit was up, so I wasn’t going on my phone right before bed. And like, I always slept better. (32)
Mindful Eating	Frequentist: Maintain H_0_ BF: Moderate evidence for H_0_	My biggest thing that I noticed was like when I’m eating meals or like kind of like waiting for something, it caused me to like kind of sit with my thoughts more, which was good. Yeah, I felt like I was like, more mindful. (05) My eating habits were, are, can be pretty crappy too. So, I was like, OK, this is one good thing I’m starting now. I’m going to pair it with like another good thing and then I’m going to try and just be better… Not just, the almost use it as like motivation… (06) When I’m working for example, we have like our hour lunch break, so typically you know I’m, I’d be on social media while I’m eating my lunch. Whereas when I had my break now I would just like solely focus on eating, which I know is, is it better thing to do? (08) I noticed like when in when I was eating, I wouldn’t go on social media as much at the same time which was kind of nice because I kind of like wolf down my food cause. (29) I felt like I was like eating better because I had, like, I don’t know, I just, like, I felt more productive. So, when you feel productive, like you want to do productive things. So, I felt like I had more time to do meal prep or, just like, make a better meal [...] (22)
Life Satisfaction	Frequentist: Reject H_0_ but small effect size BF: Anecdotal evidence for H_A_	I definitely would use the word satisfied. I felt like I was just being like better like all around. (06) And even just like the internal like, I don’t know if this is the right word, but I feel like social media in some context can be like toxic. So, I feel like not having that where you’re, like, comparing yourself to everyone or being like, oh, this person went here like, they’re so cool. They’re so much cooler than me, like, you know, just taking away that kind of negative connotation to it, I think definitely helped because when you’re present and when you’re productive and confident in what you’re doing, it makes you feel better. (28)
Stress	Frequentist: Reject H_0_ but small effect size BF: Moderate evidence for H_A_	I felt I felt normal, but like also good that I wasn’t, you know, having that much like mass media being thrown in my face and being able to just, you know, be conscious, be in the present, just get what I need to get done now rather than complaining. Oh. I have so much to do, but I don’t want to do it. I don’t know. I felt good. (13) I just felt like I had a clear head. Like I felt like I wasn’t. I didn’t have to worry about anything like through social media. It was kind of like I could focus in on one thing and not have to worry about OK, maybe someone’s posting something about whatever, right. (22)
Perceived Wellness	Frequentist: Reject H_0_ but small effect size BF: Strong evidence for H_A_	Yeah, it definitely felt better. A lot better. (14) Just cause having um like what I mentioned before, just seeing sometimes seeing like all the positive things people post on social media can be kind of um a deterrent and how you feel in daily life. So, sort of eliminating that I found in the past can sort of help my overall well-being. (19) And then but I do think like not being on my phone and not scrolling social media was helpful for my psychological like well-being in terms of … I felt like I was being productive during the day because I didn’t feel any pull to go onto my phone. I was like *I can’t use it anyways so I might as well not go on it.* (12)
Relationships	Frequentist: Reject H_0_ but small effect size BF: Anecdotal evidence for H_A_	I hung out with friends more. (12) Spending time with my family. (14) I think I spent more time with other people too. (16) Yeah, I guess I spent a little bit time with friends. My niece, I have two little nieces at home, so. (26) Spending more time with my parents, my friends, like my family, just in general. (28) I found because I still live at home with my parents. I would go upstairs and sit with my parents and just like bug them and talk to them for a little bit. (32)

*Note*. ^1^ For every health-related outcome, there were participants who did not express perceiving any changes for the specified outcome(s). Bracketed numbers represent unique numbers assigned to participants to maintain their anonymity.

## Data Availability

The data presented in this study are available on request from the corresponding author.
